# Biosynthesis of Polyunsaturated Fatty Acids in Marine Invertebrates: Recent Advances in Molecular Mechanisms

**DOI:** 10.3390/md11103998

**Published:** 2013-10-21

**Authors:** Óscar Monroig, Douglas R. Tocher, Juan C. Navarro

**Affiliations:** 1 Instituto de Acuicultura Torre de la Sal (IATS-CSIC), Ribera de Cabanes 12595, Castellon, Spain; E-Mail: jcnavarro@iats.csic.es; 2 Institute of Aquaculture, School of Natural Sciences, University of Stirling, Stirling FK9 4LA, Scotland, UK; E-Mail: d.r.tocher@stir.ac.uk

**Keywords:** biosynthesis, cephalopods, elongase of very long-chain fatty acids, fatty acyl desaturase, invertebrates, non-methylene-interrupted fatty acids, polyunsaturated fatty acid

## Abstract

Virtually all polyunsaturated fatty acids (PUFA) originate from primary producers but can be modified by bioconversions as they pass up the food chain in a process termed trophic upgrading. Therefore, although the main primary producers of PUFA in the marine environment are microalgae, higher trophic levels have metabolic pathways that can produce novel and unique PUFA. However, little is known about the pathways of PUFA biosynthesis and metabolism in the levels between primary producers and fish that are largely filled by invertebrates. It has become increasingly apparent that, in addition to trophic upgrading, *de novo* synthesis of PUFA is possible in some lower animals. The unequivocal identification of PUFA biosynthetic pathways in many invertebrates is complicated by the presence of other organisms within them. These organisms include bacteria and algae with PUFA biosynthesis pathways, and range from intestinal flora to symbiotic relationships that can involve PUFA translocation to host organisms. This emphasizes the importance of studying biosynthetic pathways at a molecular level, and the continual expansion of genomic resources and advances in molecular analysis is facilitating this. The present paper highlights recent research into the molecular and biochemical mechanisms of PUFA biosynthesis in marine invertebrates, particularly focusing on cephalopod molluscs.

## 1. Introduction

Recent special issues of this journal have highlighted the importance of the marine environment as a source of bioactive lipids (“Marine Lipids”) and of marine algae as a source of polyunsaturated fatty acids (PUFA) (“Marine Algae”) (e.g., [1]). As part of the special issue on “Marine Fatty Acids” the present article aims to build on these collections and considers marine invertebrates, especially molluscs, as potential sources of PUFA with a particular focus on recent studies investigating molecular mechanisms of PUFA biosynthesis and metabolism. In comparison to the terrestrial ecosystem, the marine ecosystem is characterized by high levels of *n*-3 long-chain PUFA (LC-PUFA; ≥C20 and ≥3 double bonds), particularly 20:5*n*-3 (eicosapentaenoic acid, EPA) and 22:6*n*-3 (docosahexaenoic acid, DHA), and consequently fish and seafood are the most important sources of these vital nutrients in the human diet [2]. Virtually all PUFA originate from primary producers but can be modified as they pass up the food chain. This is generally termed trophic upgrading and various aspects of this phenomenon have been described in recent reviews [3,4,5]. Although the main primary producers of PUFA in the marine environment are microalgae (phytoplankton), the higher trophic levels have metabolic pathways that can produce novel and possibly unique PUFA.

### 1.1. Pathways of PUFA Synthesis in Primary Producers

Primary production of PUFA in the marine environment can occur in photosynthetic microalgae, heterotrophic protists, and bacteria. *De novo* synthesis of PUFA in microalgae is largely through an aerobic pathway involving sequential addition of double bonds to saturated fatty acids, mainly 18:0 (and 16:0), via Δ9 and Δ12 (or ω6) desaturases to produce 18:2*n*-6 (linoleic acid, LA), which can then be further desaturated by Δ15 (or ω3) desaturase to give 18:3*n*-3 (α-linolenic acid, LNA) [6]. A sequence of front-end desaturases, inserting double bonds between the Δ9 bond and the carboxyl terminus, and elongases convert LNA to EPA and DHA. Conventionally this sequence is Δ6 desaturase → elongase → Δ5 desaturase → elongase → Δ4 desaturase, but in some species the initial step can be elongation to 20:3*n*-3 followed by Δ8 desaturation, and Δ17 desaturation of 20:4*n*-6 to produce EPA has also been shown to occur [6]. However, some PUFA such as 16:4*n*-3 and 18:5*n*-3, which are abundant in some classes of marine microalgae including prymnesiophytes and dinoflagellates, do not fit on the conventional pathway and, together with DHA, can be the main PUFA in these species [7]. In recent years it was discovered that PUFA could be synthesized in both prokaryotes and eukaryotes via a completely novel anaerobic pathway involving polyketide synthases (PKS) [8]. The polyketide pathway requires six enzyme proteins: 3-ketoacyl synthase (KS), 3-ketoacyl-ACP-reductase (KR), dehydrase (DH), enoyl reductase (ER), dehydratase/2-trans 3-cis isomerase (DH/2,3I), dehydratase/2-trans, and 2-cis isomerase (DH/2,2I), and consists of a defined sequence of steps adding C2 units and double bonds. Thus, the PKS pathway adds double bonds to nascent acyl chains whereas the aerobic desaturase pathway inserts double bonds into intact acyl chains. The PKS pathway was first discovered in *Shewanella* sp. that, along with *Vibrio* sp., comprise the majority of the PUFA-producing bacterial species isolated from the guts of fish and invertebrates. A review of PUFA in marine bacteria had concluded that conventional aerobic pathways must be used to synthesize PUFA [9]. However, the discovery of the PKS pathway suggests that both aerobic and anaerobic pathways can operate in marine bacteria. A similar PKS-based pathway operates in *Schizochytrium*, a thraustochytrid-like marine protist that accumulates large amounts of C22 LC-PUFA, DHA and 22:5*n*-6 [10]. The enzyme complex in *Schizochytrium* (named PUFA synthase) has sequence homology to 8 of the 11 domains of *Shewanella* PKS and comprises three genes that account for the production of DHA and 22:5*n*-6 [11]. Interestingly, a Δ4 desaturase has also been cloned from a thraustochytrid species closely related to *Schizochytrium* showing that enzymes of the aerobic pathway are also present in these organisms [12]. The PKS pathway for the production of DHA goes 16:4*n*-3 → 18:5*n*-3 → 20:6*n*-3 → DHA, while LNA, 18:4*n*-3, 20:4*n*-3 and EPA do not lie on the PKS pathway [13]. The PKS pathway may provide an explanation for the presence of 16:4*n*-3 and 18:5*n*-3 in certain microalgae and the fact that they are associated with DHA but not EPA. It may also explain the presence of PUFA > C22 such as 28:7*n*-6 and 28:8*n*-3 that have been reported in some species of marine dinoflagellates [14] as both these fatty acids lie on the PKS pathway. Although still largely speculation, these data suggest that both aerobic and anaerobic pathways may be present in different microalgal species [6,15]. Similarly, the fact that many marine bacteria produce either EPA or DHA but not both [16] may hint at differential pathways of PUFA synthesis in prokaryotes. Very recently, the marine ichthyosporean protist *Sphaeroforma arctica* was demonstrated to possess seven enzymes of PUFA biosynthesis including Δ12, ω3, Δ8, Δ5, and Δ4 desaturases, and two elongases, one with efficient elongating activity towards C18 and C20 substrates, and another with specific affinity for elongation of C20 PUFA [17].

### 1.2. LC-PUFA Biosynthesis in Fish

**Figure 1:**
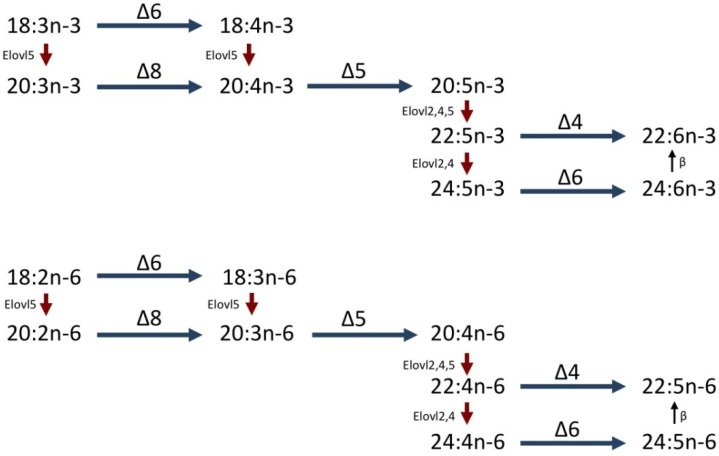
Biosynthetic pathways of LC-PUFA from C18 PUFA, α-linolenic acid (18:3*n*-3) and linoleic acid (18:2*n*-6) in vertebrates. Blue horizontal arrows represent desaturation reactions mediated by fatty acyl desaturases with Δx specificity. Red downward arrows represent elongation reactions mediated by elongases of very long-chain fatty acids (Elovl). Vertical upward arrows indicate peroxisomal β-oxidation.

Fish, like all vertebrates, cannot synthesize PUFA *de novo* from saturated and monounsaturated fatty acids and so PUFA are essential dietary nutrients [18]. However, fish do contribute to trophic upgrading and so can metabolize C18 PUFA, LNA and LA, to LC-PUFA, although whether they are able to produce EPA or DHA depends upon species. Synthesis of EPA in vertebrates is achieved by Δ6 desaturation of 18:3*n*-3 to produce 18:4*n*-3 that is elongated to 20:4*n*-3 followed by Δ5 desaturation, with synthesis of ARA from 18:2*n*-6 using the same enzymes [19] (Figure 1). In some marine fish species, the Δ6 enzyme also has Δ8 activity and so the first two steps can be reversed in order [20]. DHA synthesis from EPA requires two further elongation steps, a second Δ6 desaturation using the same Δ6 enzyme, and a chain-shortening step [21]. This pathway may operate in some species like rainbow trout (*Oncorhynchus mykiss*) [22], but recently it was shown that some species of marine fish such as rabbitfish (*Siganus canaliculatus*) and Senegalese sole (*Solea senegalensis*) have Δ4 desaturases that would enable a more direct route for the synthesis of DHA [23,24]. Therefore, the ability of a fish species to convert C18 PUFA to LC-PUFA is associated with their complement of fatty acyl desaturase (Fad) and elongase (Elovl, Elongase of very long-chain fatty acids) enzymes [13]. The molecular basis of these enzyme activities is being elucidated. This has involved cloning the cDNAs of the genes and characterising their functions by expressing the cDNAs in the heterologous yeast vector, *Saccharomyces cerevisiae*, which has no endogenous expression of LC-PUFA Fad or Elovl genes, and determining the enzyme activities through incubation of the transformed yeast with appropriate PUFA substrates. The cDNAs for Δ6 Fad have been cloned and characterized from all fish so far investigated including marine species [25]. Although a Δ6 Fad has so far not been isolated from *S. senegalensis*, nutritional and biochemical data suggest one should be present [24]. In contrast until recently, a cDNA for a discrete Δ5 Fad had only been cloned from Atlantic salmon (*Salmo salar*) [26]. Zebrafish expresses a bifunctional Δ6/Δ5 Fad [27] and a similar bifunctional Δ6/Δ5 Fad has recently been isolated from the marine herbivorous teleost, rabbitfish [23]. However, despite significant efforts, no Δ5 Fad has been found in any other marine teleost including those with sequenced genomes [28]. In mammals, several Elovl genes are known and at least two, Elovl2 and Elovl5, participate in LC-PUFA biosynthesis [29]. The cDNAs for Elovl5 have been characterized in all finfish studied, including marine species but, in contrast, Elovl2 has not been isolated from any marine fish species [25]. Functional characterization of the fish Elovls showed that Elovl5 had activity predominantly towards C18 and C20 PUFA, whereas Elovl2 had activity predominantly towards C20 and C22 PUFA. However, a further elongase, Elovl4, that predominantly elongates very long-chain PUFA > C22 in mammals [30], may be able to compensate for the lack of Elovl2 in some fish species [31]. Therefore, the varying competences of different species to biosynthesize LC-PUFA probably depends on their genome complement of both desaturase and elongase genes, with many, predominantly marine species, appearing to lack Δ5 Fad and Elovl2 elongase.

### 1.3. PUFA Production in Marine Invertebrates

The interest in LC-PUFA biosynthesis pathways in fish has been stimulated by the drive towards increasing sustainability of aquaculture through replacing the traditional major feed ingredients, fish meal and fish oil, derived from pelagic (reduction) fisheries with more sustainable alternatives such as plant meals and vegetable oils. As vegetable oils do not contain LC-PUFA, but are rich in C18 PUFA, there has been considerable research into the pathways of endogenous production of EPA and DHA in fish species [13,18,25,32]. Although algae have always been of interest as sources of LC-PUFA [6,15], they have been the subject of renewed and increased attention as genetic resources for the establishment of LC-PUFA synthesis in transgenic oilseed crops [33,34]. In contrast, little is known about the pathways of *de novo* synthesis or trophic upgrading of PUFA in the levels between the primary producers and fish, which are largely filled by invertebrates [35]. Indeed, the paradigm a couple of decades ago was that PUFA could not be synthesized by “animals” and that they could only contribute to trophic upgrading (e.g., conversion of dietary PUFA to LC-PUFA), but it has become increasingly apparent over the years that this was a gross generalization and that PUFA may indeed be able to be synthesized in lower animals [13]. The study of PUFA biosynthesis and metabolism in invertebrates has some interesting methodological challenges. As alluded to above, many marine bacteria known to produce LC-PUFA have been isolated from intestines of cold-water invertebrates. Thus, bacteria producing EPA and DHA were found in the culturable intestinal flora of 10 Arctic and sub-Arctic invertebrates [16]. In total, a third of all strains of bacteria tested contained *n*-3 PUFA with highest prevalences (>50%) from two species of bivalve *Chlamys islandica* and *Astarte* sp., and the amphipod *Gammarus wilkitzkii*. This emphasizes the importance of studying biosynthetic pathways and mechanisms at a molecular level. In this context, the present paper focuses on recent research into the molecular and biochemical mechanisms producing LC-PUFA in marine invertebrates, particularly cephalopod molluscs, that are of particular interest due to their potential as aquaculture species.

## 2. Molluscs Can Biosynthesize PUFA

Molluscs are arguably the group among marine invertebrates in which biosynthesis of PUFA has been most extensively investigated, in part due to commercial interest and perceived nutritional value of molluscs as sources of “omega-3” for humans [36]. Comprehensive reviews on the fatty acid composition of a wide variety of mollusc classes have been published previously [36,37]. A series of studies combining analytical and biochemical approaches have aimed to elucidate the ability that different mollusc species from classes including gastropods [38,39,40,41] and bivalves [42,43,44,45,46] have for endogenous production of PUFA. Generally, it is accepted that molluscs have some ability for PUFA biosynthesis but, as mentioned above for fish, such capability appears to vary among species depending upon on the enzymatic complement of desaturase and elongase enzymes involved in these metabolic reactions.

Molluscs, like other marine invertebrates [47], possess a particular group of PUFA called non-methylene-interrupted (NMI) fatty acids that can be biosynthesized endogenously [48]. While the biosynthetic pathways of NMI fatty acids have been described in detail previously [45,48] and summarized below (see Section 4), it is easy to foresee that at least two distinct desaturase activities exist accounting for the characteristic ∆5,9 unsaturation pattern of NMI fatty acids found in a wide range of mollusc species. The first is stearoyl-CoA desaturase (Scd), an enzyme universally present in living organisms [49], and confirmed to be present in molluscs [50,51,52]. Scd shows ∆9-desaturase activity and is responsible for the production of palmitoleic acid (16:1*n*-7 or ^∆9^16:1) and oleic acid (18:1*n*-9 or ^∆9^18:1), from palmitic acid (16:0) and stearic acid (18:0), respectively [53]. Second, a further desaturase with ∆5-desaturation activity is required for introducing a double bond at the ∆5 position. We recently demonstrated for the first time in molluscs that a fatty acyl desaturase isolated from the common octopus, *Octopus vulgaris*, was a ∆5-like desaturase (Section 3). Fatty acyl desaturases having ∆5-like specificity appear to be widely distributed among molluscs. Thus, two putative ∆5 desaturases were recently cloned from the abalone *Haliotis discus hannai* [54]. Functional assays in yeast confirmed the abalone desaturases had ∆5 activity and showed that they had only 53% identity to mammalian Fads [55]. In contrast, it was claimed that the Jade Tiger hybrid abalone had a ∆6 desaturase that was regulated in response to dietary fatty acid composition, although this was based solely on gene expression studies using primers based on rainbow trout ∆6 Fad sequences [56,57,58]. Consequently, while ∆6-desaturase activity was reported in some molluscs like the clam *Mesodesma mactroides* [42], the low sequence homology of the ∆5 of *H. discus hannai* with vertebrate Fads indicates that the presence of a ∆6-desaturase in Jade Tiger hybrid abalone cannot be inferred from sequence homology but rather must be confirmed through cloning and functional assays [55]. A misleading nomenclature was also used for referring to an elongase from the Jade Tiger hybrid abalone [56]. These authors reported the existence of an ‘Elongase-2’ but it is unclear whether the authors referred to this as a homolog of the vertebrate Elovl2 family.

Biochemical studies have suggested that molluscs possess elongase(s) involved in the biosynthetic pathways of PUFA including NMI fatty acids. Thus, *M. mactroides* was able to elongate 18:3*n*-3 and 18:2*n*-6 to 20:3*n*-3 and 20:2*n*-6, respectively, when maintained in seawater containing radiolabeled fatty acids [42]. Moreover, the Pacific oyster *Crassostrea gigas* had the ability to produce EPA and DHA when fed microalgae lacking these fatty acids [43], and other bivalves including *Scapharca broughtoni*, *Callista brevisiphonata*, and *Mytilus edulis* were capable of elongating the NMI dienes ^Δ5,11^20:2 and ^Δ5,13^20:2 to ^Δ7,13^22:2 and ^Δ7,15^22:2, respectively [45,46]. Further evidence of active fatty acid elongase systems in molluscs was provided by determination of fatty acid compositions in specimens subjected to different experimental regimes. The abalone *Haliotis fulgens* accumulated significant levels of elongated PUFA derived from C18 precursor fatty acids present in formulated diets containing different oil sources [59]. Elongases also accounted for the biosynthesis of unusual NMI fatty acids from the bivalve *Megangulus zyonoensis* [60]. Interestingly, these studies suggested that Δ5 desaturases were also involved in the biosynthetic pathways of some minor NMI fatty acids. Unlike desaturases, the individual enzymes responsible for the above elongation reactions have not been identified in any mollusc, with the sole exception of the common octopus elongase described below [61].

The increasing availability of DNA sequence data has revealed the presence of elongase-like genes in the genomes of several species of molluscs. Thus, *in silico* searches by the authors revealed that two transcripts encoding putative elongases with potential roles in the biosynthetic pathways of LC-PUFA in the owl limpet, *Lottia gigantea*, can be identified, one with high homology to the octopus Elovl (jgi|Lotgi|224291|), and another to Elovl4 proteins (jgi|Lotgi|178149|). Similarly, assembly of EST hits enabled us to deduce the partial amino acid sequences of Elovl-like enzymes in the molluscs *Mytilus galloprovinciallis* (gb|FL495089.1| and gb|FL499406.1|), *C. gigas* (gb|CU989853.1|, gb|HS232816.1|, gb|HS186171.1| and gb|HS245897.1|), *Euprymna scolopes* (gb|DW256301.1|), *Lymnaea stagnalis* (gb|FC701557.1|, gb|FC773093.1|, gb|FC770692.1| and gb|FC696214.1|), and *Aplysia californica* (gb|EB285681.1|, gb|GD233360.1| and gb|EB325217.1|). A similar strategy enabled us to identify sequences of putative Fads from *H.* *discus hannai* (gb|ADK38580.1| and gb|ADK12703.1|), *A. californica* (gb|XP_005090573.1| and gb|XP_005090577.1|), *C. gigas* (gb|EKC33620.1|), and *L. gigantea* (jgi|Lotgi|113523| and jgi|Lotgi|143229|). It is noteworthy that more than one Fad-encoding gene appears to exist in the genomes of some molluscs, such as *H.* *discus hannai* and *L. gigantea*, as this contrasts with previous assumptions on the presence of a single Fad gene in molluscs [62]. The continual publication of updated versions of whole-genome projects from mollusc species will help to elucidate and clarify the Fad and Elovl complements present among different mollusc classes. More importantly, functional analysis of the newly discovered genes/enzymes will be necessary to fully understand their roles in the pathways of LC-PUFA biosynthesis.

## 3. Molecular Studies on the Biosynthesis of PUFA in Cephalopods

Long-chain PUFA have been regarded as essential nutrients for cephalopods, particularly in early life-cycle stages [63]. However, which specific fatty acids were essential had not been determined due, in part, to difficulties in conducting feeding trials in species such as the common octopus (*O. vulgaris*), which experiences massive mortalities at the paralarval developmental stage [64]. In order to identify which fatty acids were essential nutrients for cephalopods, we recently conducted a series of studies aimed at the molecular and functional characterization of genes encoding specific Fad- and Elovl-like enzymes with key roles in LC-PUFA biosynthetic pathways. While the description below corresponds to investigations performed in *O. vulgaris* [61,62], this can be likely extended to the cephalopod class as very similar results were recently obtained with Fad and Elovl genes of the common cuttlefish, *Sepia officinalis* [65].

**Figure 2:**
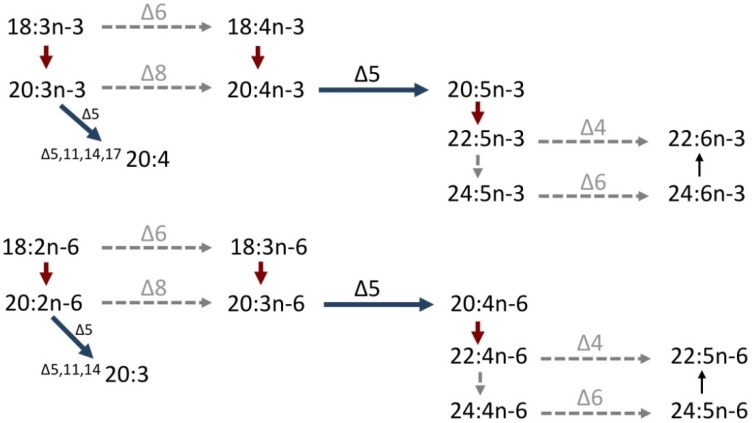
Putative PUFA biosynthetic pathways in cephalopods from C18 PUFA, α-linolenic acid (18:3*n*-3) and linoleic acid (18:2*n*-6). Solid arrows indicate demonstrated activities, whereas dashed arrows show vertebrate-based activities not determined in cephalopods. Horizontal dark blue arrows are desaturation reactions and red arrows are elongation reactions.

A cDNA encoding a front-end desaturase was isolated from *O. vulgaris* [62]. Sequence analysis indicated that the octopus Fad contained three histidine boxes (HXXXH, HXXHH and QXXHH), a putative cytochrome b_5_-like domain, and the haem-binding motif, HPGG, similar to vertebrate desaturases of the “Fads” family [53,66]. When expressed in yeast *S. cerevisiae*, the octopus Fad exhibited Δ5-desaturase activity on both saturated and polyunsaturated fatty acyl substrates. The yeast’s endogenous 16:0 and 18:0 were efficiently converted to the ∆5-desaturated monoenes 16:1*n*-11 (^∆5^16:1) and 18:1*n*-13 (^∆5^18:1), respectively. This ability of octopus Fad was consistent with the fatty acid compositions reported for other molluscs including *Littorina littorea* and *Lunatia triseriata* that showed the presence of ∆5-desaturated monoenes including 18:1*n*-13 and 20:1*n*-15 (^∆5^20:1) [36]. The ∆5-like specificity of the cephalopod Fad extended to PUFA substrates and thus 20:4*n*-3 and 20:3*n*-6 were converted to EPA and ARA, respectively (Figure 2). In contrast, no apparent ∆4, ∆6 or ∆8 activities were exhibited by the cephalopod Fad. While these results suggested that endogenous biosynthesis of both EPA and ARA from less saturated PUFA was possible in *O. vulgaris*, the biosynthetic rates *in vivo* may be of limited biological relevance due to the likely limiting availability of precursor PUFA for the Δ5 Fad. In addition to the low contents of 20:4*n*-3 and, especially, 20:3*n*-6 in natural diets of common octopus, their endogenous biosynthesis from C18 PUFA appears to also be restricted by the absence of key desaturation activities required in the initial steps of the pathways (Figure 2). Similar to the pathway described above for fish, enzymes with either Δ6- or Δ8-desaturation activities are required for the production of 20:4*n*-3 and 20:3*n*-6 from the C18 precursors LNA and LA, respectively (Figure 2). However, there is currently no evidence to support the existence of neither Δ6- nor Δ8-desaturase activities among cephalopods. It can, thus, be concluded that both EPA and ARA cannot be biosynthesized endogenously at physiologically significant rates and are therefore essential dietary nutrients for cephalopods. Similarly, the currently available molecular data suggest that DHA is also an essential fatty acid for cephalopods. First, no Δ4 desaturase enabling DHA biosynthesis from 22:5*n*-3 has been detected in cephalopods (Figure 2). Second, there is no evidence in cephalopods for the alternative, vertebrate-like, pathway involving elongation of 22:5*n*-3 to 24:5*n*-3, Δ6 desaturation to 24:6*n*-3, and chain-shortening to DHA [21], as the cephalopod Fad above does not have Δ6-activity.

We have recently confirmed the existence of a cDNA encoding an Elovl involved in the production of LC-PUFA in the cephalopod *O. vulgaris* [61]. Phylogenetic analysis comparing the amino acid sequence of the cephalopod Elovl along with other putative mollusc elongases and vertebrate elongases (Elovl1-7) showed that all the mollusc elongases, including the cephalopod Elovl, clustered as a group along at the base of the vertebrate Elovl2 and Elovl5 groups [61]. This indicated that the octopus and the other mollusc Elovl were closely related to the Elovl family members (Elovl5 and Elovl2) with well-demonstrated roles in the biosynthesis of LC-PUFA [29]. It was particularly noteworthy that the phylogenetic analysis clearly delineated the octopus and other mollusc Elovl as basal sequences to the distinct vertebrate families Elovl5 and Elovl2, and so we designate the octopus elongase as “Elovl5/2”. Functionally, however, the octopus Elovl5/2 did not show all the elongation capabilities of its vertebrate partners Elovl2 and Elovl5 [60]. More specifically, the octopus Elovl5/2 efficiently elongated C18 and C20 PUFA substrates, but had no activity towards C22 PUFA (Figure 2). Such specificity aligned with that of vertebrate Elovl5, and clearly differed from that of vertebrate Elovl2, which elongate C20 and C22, with only marginal activity on C18 PUFA [29,32,53]. Consequently, the cephalopod Elovl5/2 is not itself capable of performing the elongation of 22:5*n*-3 to 24:5*n*-3 required for DHA biosynthesis through the Sprecher pathway, and further supports the abovementioned hypothesis that endogenous biosynthesis of DHA is not possible, suggesting DHA is an essential nutrient in cephalopods. In order to investigate if other elongases could possibly complement the functions that Elovl5/2 plays in the LC-PUFA biosynthesis in cephalopods, we have recently cloned a second elongase with high homology to vertebrate Elovl4 proteins, elongases with demonstrated roles in the biosynthesis of very long-chain (C > 24) PUFA in vertebrates [30,31,67,68,69]. Functional characterization data suggest that the octopus Elovl4-like protein shows an apparent ability for the elongation of C22 PUFA substrates, thus producing polyenes with chain-lengths up to 34 carbons. However, these results require further confirmation and are not considered in the pathways depicted in Figure 2.

## 4. Molecular Mechanisms of Non-Methylene-Interrupted Fatty Acid Biosynthesis

As alluded to above, marine invertebrates are a possibly unique source of unusual PUFA, the NMI fatty acids, as opposed to the common methylene-interrupted PUFA, in that their double (ethylenic) bonds are separated by more than one methylene group. First discovered (reported) in the 1970s, these ubiquitous, although generally minor, components of the lipids of invertebrates have been extensively studied ever since (for review, see [47,48]). Although their biological role and function is not fully understood, it has been suggested that NMI fatty acids play structural and protective roles in cell membranes [48]. This hypothesis is supported by their esterification into polar lipid classes [36,70], occurrence in amounts that are often in a reverse relation to common LC-PUFA, EPA and DHA [71,72], and their selective retention in fasting animals [71]. Their presence in the body composition of filter feeders (mainly bivalves and sponges), and even predator snails [36,73,74] has often led to their association with a dietary origin. However, endogenous biosynthetic systems have been demonstrated in marine invertebrates, particularly marine sponges and molluscs [46,48,72,75]. The occurrence and biochemistry of the biosynthetic pathways of NMI fatty acids in marine invertebrates have been reviewed recently [47,48]. Here we briefly describe our recent findings on the presence of NMI fatty acids in the common octopus and discuss the molecular mechanisms likely to be involved in the biosynthetic pathways based on functional assays of the Δ5 Fad and Elovl5/2 described above [61,62].

The fatty acid composition of polar lipids prepared from adult octopus tissues (nephridium, male gonad, eye, and caecum) revealed the presence of four NMI fatty acids that were identified as ^Δ5,11^20:2, ^Δ7,13^20:2, ^Δ5,11,14^20:3, and ^Δ7,13^22:2, using GC-MS methodologies [61]. The biosynthesis of the three dienes, namely ^Δ5,11^20:2, ^Δ7,13^20:2, and ^Δ7,13^22:2, can be speculated to proceed as follows. *De novo* biosynthesis of 16:0 and 18:0 is followed by desaturation via Scd (Δ9-desaturase) to produce 16:1*n*-7 (^Δ9^16:1) and 18:1*n*-9 (^Δ9^18:1), respectively. Preliminary data have confirmed that *O. vulgaris* expresses an Scd with the necessary enzymatic abilities [76]. Subsequent elongation to 18:1*n*-7 (^Δ11^18:1) and 20:1*n*-9 (^Δ11^20:1) and further Δ5-desaturation results in the formation of ^Δ5,11^18:2 and ^Δ5,11^20:2, and a further elongation would result in the production of ^Δ7,13^20:2 and ^Δ7,13^22:2. Direct evidence for the production of ^Δ5,11,14^20:3 was obtained through functional assays on the octopus Δ5 Fad [62]. Yeast expressing the octopus Fad and grown in the presence of 20:2*n*-6 (^Δ11,14^20:2) was efficiently converted into the NMI fatty acid ^Δ5,11,14^20:3 (Figure 2). The octopus Δ5 Fad also accounted for the conversion of 20:3*n*-3 (^Δ11,14,17^20:3) to ^Δ5,11,14,17^20:4 (Figure 2), another NMI fatty acid that has been found in bivalves [44,60] and gastropods [77]. Although there is currently no direct evidence to confirm that the octopus Elovl5/2 is involved in the specific production of NMI PUFA as described above, functional characterization clearly showed it was able to elongate C18 and C20 PUFA, consistent with the above activities [61].

## 5. Biosynthesis of PUFA in Other Marine Invertebrates

### 5.1. Sponges

Fatty acids from sponges, particularly of the class Demospongiae, have unique structural characteristics that include long fatty acyl chains (up to 34 carbons), presence of branched chains and functional groups, relatively high degrees of unsaturation (up to six double bonds), and a particular pattern of unsaturation within the fatty acyl chain [48,78]. Among the great variety of NMI fatty acids detected from sponges, those with a ∆5,9 double bond pattern are the most abundant and several studies have been conducted to establish the biosynthetic pathways [47,77]. A series of elongation reactions convert 16:0 to 26:0 and it appears the two double bonds can be introduced in either order. Therefore, the first double bond can be introduced at either ∆5 or ∆9 positions, and the second double bond may be inserted on either side of the first one as necessary to produce the ∆5,9 diene. From this brief description, it is clear that active desaturation and elongation systems are present in sponges [48]. Isolation of uncommon ∆6,11 NMI fatty acids in the sponge *Euryspongia rosea* [79] revealed alternative desaturase specificities including ∆6 may also occur in some species. Unfortunately, however, no molecular studies aimed at the identification of the individual enzymes that catalyse these biosynthetic reactions have been published. *In silico* searches for putative Fad and Elovl genes in the genome of the demosponge *Amphimedon queenslandica* [80] revealed the presence of candidate enzymes involved in the LC-PUFA biosynthetic pathways in this species. More specifically, a Fad-like sequence (gb|XP_003385370.1|) and two elongases annotated as Elovl2- (gb|XP_003387250.1|) and Elovl4-like (gb|XP_003388651.1|) proteins were identified.

### 5.2. Crustaceans

Data available in the literature demonstrate that the ability of crustaceans for LC-PUFA biosynthesis varies among species, with no obvious patterns associated with phylogenetics (classes/subclasses) or habitat (freshwater/marine). For example, harpacticoid copepods, but not calanoid species, have the ability to biosynthesize LC-PUFA, possibly related to poor quality of food available in detritus-rich habitats. Thus, the presence of desaturases and elongases involved in the biosynthesis of PUFA have been demonstrated in harpacticoid copepods [81,82,83,84,85]. Similarly, biochemical and analytical data indicated that the freshwater cyclopoid copepod *Eucyclops serrulatus* had the ability to endogenously produce DHA when fed on microalgae devoid of this fatty acid [86]. On the contrary, calanoid copepods appear to be unable to endogenously synthesize LC-PUFA at physiologically significant rates, similar to other species of marine zooplankton including *Dropanopus forcipatus* and *Euphausia superba* [13,87,88].

While the ability for endogenous LC-PUFA biosynthesis in some crustaceans has often been indicated through indirect analytical (compositional) approaches, more direct (biochemical and/or molecular) evidence demonstrating the above mentioned enzymatic activities are scarce and thus the specific biosynthetic pathways remain elusive. Recent investigations have shown the existence of a putative desaturase from the Chinese mitten crab, *Eriocheir sinens* [89,90]. While representing pioneer molecular studies on the biosynthesis of PUFA in crustaceans, no functional analyses were presented, with gene/protein annotation as Δ9- and Δ6-like desaturases being based on phylogenetic analysis alone. It is necessary to emphasize that assessment of the actual enzyme activity should be determined before function is ascribed. This is particularly critical in species in which the precise LC-PUFA biosynthetic pathways are likely to vary with respect to the accepted vertebrate model. However, the Chinese mitten crab desaturases were shown to be regulated through diet and increased expression was observed in individuals fed on diets formulated with relatively high contents of vegetable oil (soybean oil), similarly to well-established regulatory responses in desaturase expression observed in vertebrate species including fish [32]. This result may suggest a putative role for the desaturases in the biosynthesis of PUFA in *E. sinens*.

Some ability for endogenous production of LC-PUFA has also been reported in other crustaceans including the branchiopoda *Daphnia pulex*, which showed increased LC-PUFA content in response to low temperature exposure [91], and *Artemia*, with an apparent ability to interconvert fatty acids from the *n*-6 to *n*-3 series. [92,93]. The latter studies, in which radiolabeled LA (18:2*n*-6) was converted to LNA (18:3*n*-3), were particularly interesting as they implied the existence of an *n*-3 (or Δ15) desaturase in crustaceans, enzymes that were believed to be absent in animals but now confirmed to exist in some invertebrates like the nematode *C. elegans* [94,95]. Interestingly, BLAST searches revealed the existence of a sequence with high homology to *n*-3 desaturases in the copepod *Caligus rogercresseyi* (gb|ACO10720.1|), an ectoparasite that causes major issues in the Atlantic salmon farming industry. In addition, within this species, other sequences for potential candidate enzymes were identified including desaturases (gb|ACO10922.1), annotated as “Delta-5 fatty acid desaturase”, and elongases homologous to vertebrate Elovl2 (gb|ACO10776.1|) and Elovl4 (gb|ACO11542.1|).

### 5.3. Cnidarians

The presence of other organisms, including primary producers, within some invertebrates, which complicates the unequivocal identification of PUFA and/or LC-PUFA biosynthetic pathways, is particularly pertinent when considering cnidarians that have symbiotic interactions with dinoflagellate microalgae (zooxanthellae) that are able to synthesize PUFA [96]. Furthermore, fatty acid translocation has been reported or implied to occur from endosymbiotic dinoflagellates into various host organisms including tridacnid bivalve molluscs (giant clams) [97], and cnidarians including anthozoans (corals and sea anemones) [98] and scyphozoans (jellyfish) [99]. Thus, active transport of saturated and unsaturated fatty acids from zooxanthellae to the host has been reported, and lipids from symbiosis can account for up to 46% of coral tissue dry weight [100,101]. Therefore, fatty acid compositions of polytrophic organisms like corals will reflect the derivation of nutrients from various sources, including zooxanthellae and other endogenous algae and bacteria, and fatty acids can serve as markers of these sources [102]. Analysis of the distribution of PUFA in zooxanthellae, polyp tissue, and intact colonies in soft coral *Sinularia* sp. and hard coral *Acropora* sp. provided clues to what biochemical pathways of PUFA synthesis were present in the different fractions [103]. The results showed that 18:3*n*-6, 18:4*n*-3, EPA, 22:5*n*-3 and DHA were mainly synthesized by the zooxanthellae, and that 20:3*n*-6, ARA, and 22:4*n*-6 were synthesized in the polyp tissue. Soft coral polyps were also able to synthesize 24:5*n*-6 and 24:6*n*-3. These tetracosapolyenoic fatty acids are chemotaxonomic markers of soft corals [102], regardless of the presence or not of zooxanthellae [104], showing that their biosynthesis from C22 PUFA occurs only in the coral polyps. The zooxanthellae also synthesized 16:2*n*-7, 16:3*n*-4, and 16:4*n*-1. The authors speculated that the biosynthesis of 16:2*n*-7 in *Sinulari*a sp. and 18:3*n*-6 in *Acropora* sp. was catalyzed by a Δ6 desaturase, and that the relatively even distribution of 18:2*n*-6, 18:3*n*-6, and 16:2*n*-7 among the different fractions indicated translocation between zooxanthellae and coral polyps [103]. Consistent with this, the polyps synthesized 18:2*n*-7, perhaps suggesting the action of a C16 elongase in the coral. In contrast, a recent study with the model anemone, *Aiptasia pulchella*, using enriched stable isotopic (^13^C) incorporation from dissolved inorganic carbon, showed fatty acid synthesis rates were attributed to only a complex integration of lipogenesis pathways within the dinoflagellate symbionts, and that there was no evidence of symbiont-derived enriched isotope fatty acids being directly utilized in host PUFA synthesis [105].

### 5.4. Other Non-vertebrate Groups

Little is known about PUFA biosynthesis and metabolism in tunicates, formerly known as urochordates. However, embryonic development in ascidian tunicates (sea squirts) is simple, rapid, and easily manipulated which, along with transparency, has made them suitable models for studying the fundamental developmental processes of chordates. As a result, good genomic resources are available and, indeed, the genome of the sea squirt, *Ciona intestinalis*, was one of the first published a decade ago. A search of the *C. intestinalis* genome using the Δ6 elongase sequence from the moss *Physcomitrella patens* as a query, indicated the presence of an elongase, and functional characterization of the ORF in the yeast *S. cerevisiae* revealed it was capable of elongating both 18:4*n*-3 and EPA [106]. However, it is not know if these activities are representative of their functional role in this tunicate.

As mentioned previously, increasing availability of genomic data from invertebrate organisms is a useful tool for the identification of possible desaturase and elongase genes with potential roles in the biosynthesis of PUFA and/or LC-PUFA. Thus, some desaturases with high homology to vertebrate Fads-like proteins, and to the ∆5-like desaturases from molluscs, can be found in the GenBank public database. These include desaturases in *C. intestinalis* (gb|NP_001029014.1|), the polychaete worm (Annelida) *Capitella teleta* (gb|ELT94279.1|), and the Acorn worm (Hemichordata) *Saccoglossus kowalevskii* (gb|XP_002739666.1), among others. Putative Elovl2- and Elovl5-like elongases include those from the polychaete *C. teleta* (ELU02248.1) and the purple sea urchin (Echinodermata) *Strongylocentrotus purpuratus* (gb|XP_789039.2|).

## 6. Concluding Remarks

Almost all PUFA originate in microalgae, bacteria and heterotrophic protists inhabiting aquatic ecosystems. Fish, occupying relatively high trophic levels within aquatic systems, can significantly contribute to trophic upgrading as they have the ability to metabolize PUFA to produce LC-PUFA. However, the pathways of PUFA biosynthesis in invertebrates, organisms occupying trophic levels between the primary producers and fish in aquatic ecosystems, have remained largely unexplored. Particularly challenging in the study of fatty acid metabolism in invertebrates is the potential presence of microflora and symbiotic organisms with active metabolic pathways that can hinder the unequivocal assignment of enzymatic activities to the host invertebrate. Molecular studies clearly emerge as a valuable strategy to address this challenge and, thus, significant progress is now being made to understand the biosynthetic pathways and mechanisms producing LC-PUFA in marine invertebrates.

Data available in the literature provide evidence that marine invertebrates possess and express genes encoding desaturase and elongase enzymes with a role in the endogenous production of LC-PUFA. Ever-increasing genomic data derived from sequencing projects conducted for a wide range of marine invertebrates is providing an excellent source of molecular evidence supporting the existence of these biosynthetic pathways. While potential candidates for both desaturase and elongase genes have been identified in sponges, cnidarians, annelids, echinoderms, hemichordates, and tunicates, a more robust set of data combining analytical, biochemical and molecular evidence is available for crustaceans and, particularly, molluscs. Using the common octopus as model species, we have begun to elucidate pathways for the biosynthesis of PUFA in cephalopods and possibly other molluscs. To this end, several genes encoding desaturases and elongases that are directly involved in the production of PUFA have been identified by molecular cloning, and functionally characterized by heterologous expression in yeast. A Δ5 desaturase has been confirmed in the cephalopods, octopus and cuttlefish [62,65], and in the gastropod, abalone [54] and, thus, appears to be a common enzyme component involved in PUFA biosynthetic pathways in molluscs. Furthermore, although not yet demonstrated at a molecular level, the ubiquitous distribution of Δ5,9 NMI fatty acids (as well as their corresponding elongation products) among marine invertebrates including sponges, cnidarians, and echinoderms [47] suggests the presence of Δ5-like desaturases in these groups. In addition, the biosynthesis of Δ5,9 NMI fatty acids implies the action of a Δ9 desaturase, an enzymatic activity of Scd, a desaturase universally distributed in all living organisms and confirmed in the common octopus [76]. Other desaturases involved in LC-PUFA pathways have been hypothesized to exist in some invertebrate groups. Thus, the sponge *Euryspongia rosea* [79] was found to contain uncommon ∆6,11 NMI fatty acids of which production would suggest the action of a ∆6-desaturase. Additionally, some crustaceans appear to express an *n*-3 (or Δ15) desaturase, an enzyme catalyzing the interconversion of *n*-6 to *n*-3 fatty acids [92,93]. Further studies are required to determine the distribution of these desaturases among marine invertebrates. More importantly, these studies must be accompanied by functional characterization analyses that confirm the precise activities of the enzyme.

Fatty acid elongation systems have also been described in marine invertebrates. *In silico* searches for Elovl-like sequences in the genome of marine invertebrates indicate that homologs to vertebrate Elovl2, Elovl4, and Elovl5 exist. Interestingly, while the proteins are distinguished as three distinct families in vertebrates [29], only two appear to be present in invertebrates. Thus, in addition to an Elovl4-like sequence, cephalopods appear to possess a single Elovl5/2 protein that is basal to the distinct vertebrate families Elovl5 and Elovl2. Such Elovl5/2 basal proteins are also present in the demosponge *Amphimedon queenslandica*, the copepod *Caligus rogercresseyi*, the polychaete *Capitella teleta*, and the purple sea urchin *Strongylocentrotus purpuratus*. Functionally, the cephalopod Elovl5/2 is similar to that of vertebrate Elovl5, with ability to efficiently elongate C18 and C20 PUFA substrates, but no activity on C22 PUFA. These results reveal an interesting evolutionary scenario predicting the divergence of the Elovl5/2 basal protein into distinct protein families in vertebrates. Ongoing investigations in *O. vulgaris* suggest that the cephalopod Elovl4-like protein participates in the biosynthesis of very LC-PUFA (34 carbons).

Studies of the molecular mechanisms underlying PUFA and LC-PUFA biosynthesis in marine invertebrates are not only illuminating alternative and unusual biosynthetic pathways and metabolism, but are also providing insights to gene and pathway evolution, as well as being a resource that can supply potentially valuable molecular tools in the form of genes involved in PUFA biosynthesis and metabolism.
